# Hyperprogressive disease rarely occurs during checkpoint inhibitor treatment for advanced melanoma

**DOI:** 10.1007/s00262-020-02716-3

**Published:** 2020-09-14

**Authors:** M. Schuiveling, E. H. J. Tonk, R. J. Verheijden, K. P. M. Suijkerbuijk

**Affiliations:** Department of Medical Oncology, University Medical Center Utrecht Cancer Center, Utrecht, The Netherlands

**Keywords:** Melanoma, Immune checkpoint inhibitors, Hyperprogression, Hyperprogressive disease, Anti-PD1

## Abstract

**Introduction:**

Hyperprogression, characterized by a rapid acceleration in tumor growth, is a novel pattern of progression recently described in patients treated with immune checkpoint inhibitors. This study aims to assess the incidence of hyperprogression in patients with advanced melanoma treated with checkpoint inhibitors.

**Methods:**

Clinical and radiological findings of all advanced melanoma patients who started checkpoint inhibitors between January 2013 and March 2019 in a tertiary academic center in the Netherlands were analyzed. Change in tumor burden was calculated by assessing volumetric tumor growth using the criteria as defined by immune Response Evaluation Criteria in Solid Tumors version 1.1. Hyperprogression was defined as a time to treatment failure less than 2 months with doubling of tumor burden and a twofold increase in tumor growth rate during treatment. Possible hyperprogression was defined as the presence of the first two criteria in the absence of a pre-baseline scan.

**Results:**

Out of 206 treatment episodes in 168 patients, 75 were evaluable for hyperprogression and 87 for possible hyperprogression. Hyperprogression was observed in one patient (1.3%) and possible hyperprogression was observed in one patient (1.1%).

**Conclusion:**

Hyperprogression is rare in melanoma patients treated with immune checkpoint inhibitors. Our data question if hyperprogression really is a biological entity in metastatic melanoma.

## Introduction

Over the last decade, treatment of metastatic melanoma has profoundly improved due to the introduction of immune checkpoint inhibitors (ICI) against cytotoxic T-lymphocyte-associated protein 4 (CTLA4) and programmed cell death 1 (PD1). Anti-CTLA4 re-induces the T-cell activation by antigen presenting cells whereas anti-PD1 inhibits the interaction leading to tumor escape between T-cells and tumor cells in the tumor microenvironment [1, 2].

The introduction of ICI led to the observation of novel tumor treatment responses, such as a delayed response or pseudoprogression [3]. Moreover, recent studies reported hyperprogression, an unprecedented acceleration in tumor growth during treatment with PD1/PD-L1 inhibitors [4–7]. In the consensus reached at the 2019 annual meeting of the American Association of Cancer Research (AACR), hyperprogression was defined as a time to treatment failure (TTF) of less than 2 months with a twofold increase in disease progression and doubling of the patients tumor burden compared with pre-baseline imaging [8]. Recently, studies have reported an incidence rate of 9–43% using different definitions of hyperprogression [4–7]. In melanoma, hyperprogression was observed in 9% out of 45 patients analyzed in a retrospective cohort [4]. In addition, hyperprogression was described in 43% of 51 patients with mucosal and acral melanoma [9].

Several mechanisms have been put forward as the pathophysiological drive behind hyperprogression. It has been postulated that anti-PD1 treatment can induce the proliferation of regulatory T-cells in the tumor microenvironment resulting in inhibition of anti-tumor immunity [10]. In addition, there is evidence suggesting that ICI-mediated inhibition of novel expressed PD1 on non-small-cell lung carcinoma tumor cells results in induction of tumor growth [11]. Furthermore, it has been hypothesized that in hyperprogression, anti-PD1 therapy leads to tumor infiltration of immunosuppressive M2-like macrophages [12].

Despite the growing amount of retrospective cohort studies reporting hyperprogression as a new pattern of progression in different types of cancers, some clinicians believe that the reported rapid progression in hyperprogression is just a subset of the natural course of malignant disease [8]. To substantiate the existence of hyperprogression, several studies compared ICI therapy with conventional chemotherapy. In these studies, a higher rate of hyperprogression was found in patients treated with anti-PD1 therapy [5, 13]. To date, no large study has been performed analyzing hyperprogression using the AACR criteria in advanced melanoma. Therefore, we evaluated the incidence of hyperprogression in a retrospective cohort of melanoma patients treated with ICI in an academic center in the Netherlands.

## Patients and methods

### Patients

Data from consecutive patients treated with anti-PD1, anti-CTLA4 or anti-PD1 + anti-CTLA4 for advanced melanoma from January 2013 (after introduction and reimbursement of ipilimumab in the Netherlands late 2012) until March 2019 at the University Medical Center in Utrecht were collected. The patients were included if measurable disease according to Response Evaluation Criteria in Solid Tumors (RECIST) 1.1 was present on a baseline computed tomography (CT) scan or magnetic resonance imaging (MRI) scan [14, 15]. The patients were excluded if no baseline scan was made within 6 weeks before ICI treatment or within 24 h after start of treatment or if no follow-up scan had been made within 16 weeks after the start of treatment. Since ICI are used as first-line treatment in advanced melanoma, many patients did not have a pre-baseline scan. To be able to assess whether these patients could have experienced hyperprogression, we did not exclude patients for unavailability of a pre-baseline scan.

All scans were reviewed by a radiologist according to standard institutional practice. If target lesions had not been defined previously, they were retrospectively measured by one of the investigators (M.S.). Clinicopathological characteristics, including age, gender, melanoma stage (7th edition of the American Joint Committee on Cancer), treatment type, immune-related adverse events, performance status, previous therapy and serum lactate dehydrogenase (LDH), C-reactive protein and complete blood cell count at the start of treatment and radiological evaluation were collected from all patients.

### Definition of hyperprogression

Hyperprogression was defined according to the criteria set at the 2019 AACR Annual Meeting. If no pre-baseline scan was available, TTF less than 2 months and a doubling of volumetric tumor burden in the 2 months period were used to define possible hyperprogression. Target lesions, measured according to RECIST 1.1, were used for calculation of tumor growth rate (TGR) and tumor burden. Response to treatment was evaluated according to iRECIST 1.1 criteria. Volumetric tumor burden was calculated according to the method previously described by Champiat et al. [4] A twofold increase in tumor growth was calculated by dividing the TGR during the ICI treatment period with the pre-baseline reference period [4]. Doubling of tumor burden in 2 months corresponded with TGR ≥ √2 per month.

## Results

### Description of the cohort

**Figure 1:**
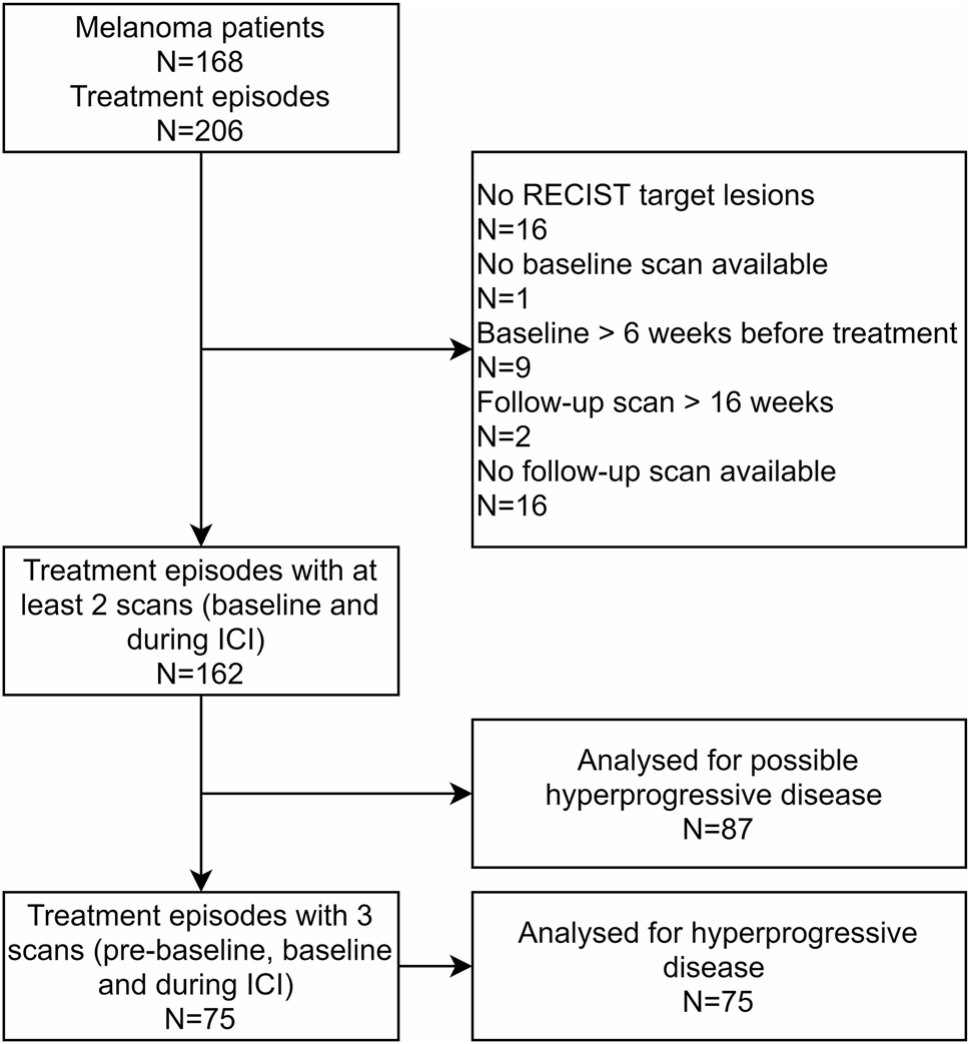
Flowchart of study selection process

A total of 168 advanced melanoma patients treated with ICI were analyzed. Due to multiple treatment episodes in 38 patients, 206 treatment episodes were evaluable. Treatment episodes were excluded for analysis in 44 patients. Main reasons for exclusion were the absence of target lesions (*N* = 16) and the unavailability of a follow-up scan (*N* = 16). Follow-up scans were absent due to rapid clinical deterioration after progression under targeted therapy with BRAF and/or MEK inhibitors (*N* = 5), due to clinical deterioration in patients with preexisting aggressive disease (*N* = 5), due to treatment cessation because of cerebral hemorrhage which is common in patients with melanoma brain metastases (*N* = 5), and due to a patient refusing further radiological evaluation because of claustrophobia (*N* = 1) (Fig. 1). The patient’s characteristics are visualized in Table 1.

**Table 1:** Treatment episodes characteristics stratified according to hyperprogressive disease

Treatment episodes	All (*N* = 162)	Non-hyperprogression (*N* = 160)	(Possible) hyperprogression (*N* =2)
**Gender**			
Male	99 (61%)	97	2
Female	63 (39%)	63	0
**Melanoma stage**^a^			
IIIC	3 (2%)	3	0
IV M1a	28 (17%)	28	0
IV M1b	19 (12%)	19	0
IV M1c	112 (69%)	110	2
**Mutational status**			
BRAF mutant	85 (52%)	84	1
KIT mutant	2 (1%)	2	0
NRAS mutant	30 (19%)	29	1
BRAF, NRAS mutant	3 (2%)	3	0
Wildtype	41 (25%)	41	0
**Treatment type**			
Anti-PD1	92 (57%)	91	1
Anti-PD1 + Anti-CTLA4	28 (18%)	27	1
Anti-CTLA4	42 (26%)	42	0
**Line of systemic therapy**			
First	98 (60%)	97	1
Second	51 (31%)	50	1
Third	11 (7%)	11	0
Fourth	2 (1%)	2	
**Toxicity grade 3 or higher**			
Present	39 (24%)	39	0
Absent	123 (76%)	122	2
**WHO Performance status**			
0–1	142 (88%)	141	1
≥ 2	18 (11%)	17	1
Missing	2 (1%)	2	0
**Previous radiation therapy**			
Yes	53 (33%)	52	1
No	109 (67%)	108	1
**Previous chemotherapy**			
Yes	16 (10%)	16	0
No	146 (90%)	144	2
**Previous ICI**			
Yes	31 (19%)	31	0
No	131 (81%)	129	2
**Previous targeted therapy**			
Yes	33 (20%)	32	1
No	129 (80%)	128	1
**Metastatic sites**			
0–2	70 (43%)	70	0
> 3	92 (57%)	90	2
**Liver metastasis**			
Present	49 (30%)	48	1
Absent	113 (70%)	112	1
**LDH at start ICI** ^b^			
> 2x Upper limit of normal	15 (9%)	14	1
≤ 2x Upper limit of normal	141 (87%)	140	1
Missing	6 (4%)	10	0

*PD1* programmed cell death 1, *PD-L1* programmed cell death ligand 1, *CTLA4* cytotoxic T-lymphocyte-associated protein 4, *LDH* lactate dehydrogenase, *ICI* immune checkpoint inhibitor

^a^7th edition of melanoma staging of the American Joint Committee on Cancer

^b^Upper limit of normal = 250 U/L

Best overall response by iRECIST was complete response in 17(10%) episodes, partial response in 45(28%) episodes, stable disease in 32(20%) episodes, unconfirmed progressive disease in 34(21%) episodes and confirmed progressive disease in 34(21%) episodes.

### Assessment of hyperprogression

**Figure 2:**
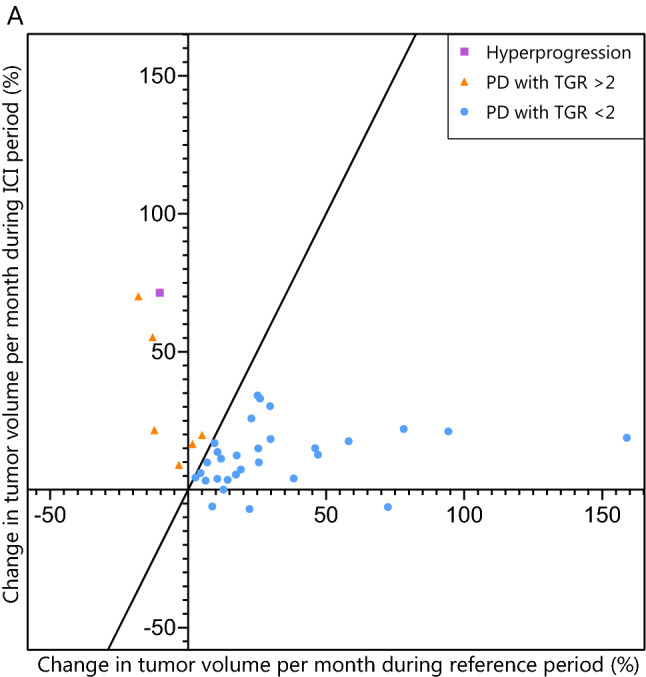
Scatterplot of change in estimated tumor volume in treatment episodes with progressive disease. Pairwise comparison of TGR between the reference and ICI treatment period in the 42 episodes with progressive disease and three evaluable radiological scans. The purple dot represents an episode with hyperprogression (2-fold increase in TGR with TTF in 2 months and 50% increase in tumor burden), the orange dots represent episodes with progressive disease and a 2-fold increase in TGR and the blue dots represent episodes with progressive disease without a 2-fold increase in TGR. *TGR* tumor growth rate, *ICI* immune checkpoint inhibitor, *PD* progressive disease by RECIST at first evaluation, *TTF* time to treatment failure

Seventy-five treatment episodes were evaluable for analysis of hyperprogression. The remaining 87 melanoma episodes did not have an evaluable pre-baseline scan and were only evaluable for possible hyperprogression. In seven out of 75 melanoma episodes, a twofold increase in TGR was observed (Fig. 2). Out of the seven episodes with a twofold increase in volumetric tumor growth, only one (1.3%) met the other two criteria for hyperprogression. In this case, hyperprogression occurred after the patient had switched to ICI for progressive disease on vemurafenib, a BRAF-inhibitor [16].

The remaining 87 melanoma episodes were analyzed for possible hyperprogression. Three episodes had a TTF less than 2 months and a 50% increase in tumor burden in 2 months. In two of these episodes, a pre-baseline scan, made between 1 week or 2 months before treatment initiation, respectively, was present. These pre-baseline scans were not included for the evaluation of pre-baseline TGR due to the absence of measurable target lesions. However, a substantial tumor load was present at the baseline scan indicating aggressive tumor behavior before start of ICI. The aggressive tumor growth during the pre-ICI treatment period makes a twofold increase in tumor growth during ICI treatment very unlikely, and thus we did not consider these patients as having possible hyperprogressive disease. In the only treatment episode defined as possible hyperprogression (1.5%), a pre-baseline scan was not present. This patient had extensive hepatic, pulmonary and bone metastatic disease with a LDH of 1822 U/L at start of ICI therapy, indicative of aggressive tumor biology.

## Discussion

In this study, hyperprogression or possible hyperprogression was observed in 2 out of 162 treatment episodes (1.2%) in 142 advanced melanoma patients treated with ICI. Hyperprogression was observed in one patient treated with anti PD-1 whereas possible hyperprogression was observed in a patient treated with anti-PD1 + anti-CTLA4.

To the best of our knowledge, this is the first large retrospective cohort study evaluating the incidence of hyperprogression in melanoma patients treated with ICI. Further analysis of the observed hyperprogression case leads to a possible alternative explanation for the rapid increase in tumor growth. Hyperprogression was observed after the patient had progressed on BRAF inhibition. Previous research shows that treatment initiation with anti-PD1 ICI following treatment failure with BRAF inhibitors is often followed by rapid disease progression (median progression free survival 2.6 months) [17]. This is also seen in our cohort in which, in addition to the hyperprogression case, both non-hyperprogression treatment episodes with a 50% increase in volumetric tumor growth per month and a twofold increase in tumor growth experienced previous disease progression on a BRAF inhibitor. In the case of possible hyperprogression, a high baseline serum LDH and diffuse metastatic spread were observed, which might suggest aggressive tumor biology before the initiation of anti-PD1 + anti-CTLA4. As alternative explanations exist for the observed rapid tumor growth for both episodes of hyperprogression and possible hyperprogression, one can question if hyperprogression is really a biological entity in metastatic melanoma.

To date, different definitions have been used to define hyperprogression resulting in widely spread incidence rates. Champiat et al. report an incidence of hyperprogression of 9% (4/45 patients) during the treatment of melanoma with anti-PD1 in phase 1 trials [4]. In their study, hyperprogression was defined as a twofold increase in volumetric tumor growth only, which does not take TTF or an increase in tumor burden into account. This could have led to overestimation of the hyperprogression rate. Besides, Champiat et al. analyzed data from phase 1 clinical trials, including heavily pretreated patients who might be more prone to rapid progression.

Forschner et al. reported an incidence of hyperprogression of 43% (22/51 patients) in anorectal and acral melanoma patients, which is remarkably higher compared to our results [9]. In this study, hyperprogression was defined as an increase in tumor load of > 50% at first staging while not taking pre-ICI tumor growth into account. Due to this lower threshold, it is unclear whether the high incidence reported by Forschner et al. reflects true hyperprogression instead of natural disease progression.

In order to avoid different definitions being used, the AACR defined consensus criteria. By requiring a substantial increase in tumor burden combined with an increase in TGR, the AACR criteria provide tools to distinguish hyperprogression from natural disease progression [8]. Recent research showed that the AACR criteria are the only criteria able to define a difference in overall survival between progressive disease and hyperprogression in non-small-cell lung carcinoma, further strengthening the ability of the AACR criteria to define true hyperprogression [18].

Our study has some limitations. The retrospective nature of this analysis makes it prone to bias. By excluding patients lacking a follow-up scan, selection bias might have occurred, although the number of patients lacking a follow-up scan (9.9%) is similar to that of Champiat et al. (8.3%) and lower than that of Forschner et al (73%). As indicated, some of these patients experienced rapid clinical deterioration, in which case we cannot completely rule out the occurrence of hyperprogression. However, most patients without a follow-up scan had preexisting aggressive disease or progressive disease after previous targeted therapy which presumably explains the rapid clinical deterioration before a follow-up scan was made.

## Conclusion

In our cohort, we identified possible hyperprogression in less than 2% of checkpoint inhibitor treated advanced melanoma patients using AACR consensus criteria for hyperprogression. Our data question if hyperprogression is really a biological entity in metastatic melanoma.

## Data Availability

The datasets generated during and/or analyzed during the current study are available from the corresponding author on reasonable request.

## Abbreviations

AACR: American Association of Cancer Research
CRP: C-reactive protein
CTLA4: Cytotoxic T-lymphocyte-associated protein 4
CT-scan: Computerized tomography scan
ICI: Immune checkpoint inhibitor
iRECIST: Immune Response Evaluation Criteria in Solid Tumors
LDH: Lactate dehydrogenase
MRI-scan: Magnetic resonance imaging scan
PD1: Programmed cell death 1
TGR: Tumor growth rate
TTF: Time to treatment failure
